# Outstanding Characteristics of Birt–Hogg–Dube Syndrome in Korea

**DOI:** 10.3390/diagnostics13122047

**Published:** 2023-06-13

**Authors:** Hye Jung Park, Yong Jun Choi, Chul Hwan Park, Tae Hoon Kim, Sung Soo Lee, Duk Hwan Moon, Kyung-A Lee, Sang Eun Lee, Moo Suk Park, Song Yee Kim, Yoon Soo Chang, Seok Jeong Lee, Ji Ye Jung, Ji-Ho Lee, Su Hwan Lee, Taehee Kim, Sung-Ryeol Kim, Kangjoon Kim, Min Kwang Byun

**Affiliations:** 1 Department of Internal Medicine, Gangnam Severance Hospital, Yonsei University College of Medicine, Seoul 06273, Republic of Korea; craft7820@yuhs.ac (H.J.P.);; 2 Department of Radiology, The Research Institute of Radiological Science, Gangnam Severance Hospital, Yonsei University College of Medicine, Seoul 06273, Republic of Korea; 3 Department of Thoracic Surgery, Yonsei University College of Medicine, Seoul 06273, Republic of Korea; 4 Department of Laboratory Medicine, Gangnam Severance Hospital, Yonsei University College of Medicine, Seoul 06273, Republic of Korea; 5 Department of Dermatology, Gangnam Severance Hospital, Yonsei University College of Medicine, Seoul 06273, Republic of Korea; 6 Division of Pulmonary and Critical Care Medicine, Department of Internal Medicine, Severance Hospital, Yonsei University College of Medicine, Seoul 06273, Republic of Korea; 7 Department of Internal Medicine, Yonsei University Wonju College of Medicine, Wonju 26426, Republic of Korea; 8 Division of Pulmonary, Allergy, and Critical Care Medicine, Department of Internal Medicine, Hallym University Kangnam Sacred Heart Hospital, Hallym University College of Medicine, Seoul 07442, Republic of Korea; 9 Division of Pulmonology, Allergy and Critical Care Medicine, Department of Internal Medicine, Yongin Severance Hospital, Yonsei University College of Medicine, Seoul 06273, Republic of Korea

**Keywords:** Birt–Hogg–Dube syndrome, cystic lung disease, chest computed tomography, folliculin gene (FLCN) mutation

## Abstract

Birt–Hogg–Dube (BHD) is a rare genetic disorder characterized by multiple lung cysts, typical skin manifestations, and renal tumors. We prospectively enrolled thirty-one subjects from four South Korean institutions with typical lung cysts, and next-generation sequencing was conducted. We prospectively enrolled thirty-one subjects from four Korean institutions with typical lung cysts. Next-generation sequencing was performed to investigate mutations in the following genes: FLCN, TSC1, TSC2, CFTR, EFEMP2, ELN, FBLN5, LTBP4, and SERPINA1. BHD was diagnosed in 11 of the 31 enrolled subjects (35.5%; FLCN mutations). Notably, we identified three novel mutations (c.1098G>A, c.139G>T, and c.1335del) that have not been previously reported. In addition to FLCN mutations, we also observed mutations in CFTR (16.1%), LTBP4 (9.7%), TSC2 (9.7%), TSC1 (3.2%), ELN (3.2%), and SERPINA1 (3.2%). According to a systematic review of 45 South Korean patients with BHD, the prevalence of pneumothorax (72.7%) was greater in South Korea than in the rest of the world (50.9%; *p* = 0.003). The prevalence of skin manifestations (13.6%) and renal tumors (9.1%) was lower in Korea than in the rest of the world, at 47.9% [*p* < 0.001] and 22.5% [*p* = 0.027], respectively). This study confirmed a significant prediction model for BHD based on age, number of lung cysts (>40), and maximal diameter of lung cysts (>2 cm) regardless of skin manifestations and renal tumors. Importantly, three novel mutations (c.1098G>A, c.139G>T, and c.1335del) were identified. In conclusion, South Korean patients with BHD display characteristics that are different from those observed in patients of other nationalities. Detailed characterization of lung cysts is needed to define BHD, especially in South Korea, even if patients do not present with skin or renal lesions.

## 1. Introduction

Although Birt–Hogg–Dube syndrome (BHD) is a rare genetic disorder, studies have revealed that BHD is increasingly being diagnosed [1]. As BHD is caused by mutations in the folliculin gene (*FLCN*), which is a tumor suppressor gene, it can be fatal [2,3]. Patients with BHD can develop cancer in various organs [4]. Inheritance occurs in an autosomal dominant manner; therefore, its early detection can save the lives of those affected or their families. The major diagnostic criteria for BHD, suggested by Menko et al. in 2009, are: (1) skin manifestations (fibrofolliculomas or trichodiscomas) and (2) *FLCN* mutation; minor criteria (1) multiple lung cysts, (2) renal cancer, and (3) a first-degree relative with BHD [1]. Clinicians screen subjects for BHD when skin manifestations, lung cysts, and/or renal cancer are present. 

A prospective study of six patients with BHD conducted by the current authors showed that the participants did not present with typical skin and renal lesions [5]. Another recent South Korean study reported a relatively low incidence of skin and renal lesions in patients with BHD [6,7]. Recent studies have reported less frequent skin and kidney involvement in the symptoms of BHD subjects in East Asia [8]. We assumed patients in Korea are at a high risk for BHD if they have multiple lung cysts located bilaterally and basally, even if skin or renal lesions are absent. However, none of studies so far have systematically reviewed Korean patients with BHD. Moreover, whether detailed characterization of lung cysts, regardless of skin and renal lesions, can predict BHD, remains unknown.

This study aimed to reveal the typical characteristics of BHD in South Korea. Additionally, we assessed the power of a prediction model that did not consider skin and renal lesions for BHD.

## 2. Methods

### 2.1. Ethics Approval and Patient Consent 

This study was approved by the Institutional Review Board of Gangnam Severance Hospital, Yonsei University Health System (approval number: 3-2018-0317).

### 2.2. Patient and Public Involvement

Patients and the public were not involved in the design, or conduct, or reporting, or dissemination plans of our research.

### 2.3. Patients 

We prospectively enrolled 31 subjects who attended Gangnam Severance Hospital, Severance Hospital, Yongin Severance Hospital, and Wonju Severance Hospital from June 2019 to May 2021 with typical lung cysts concordant with the minor criteria for the diagnosis of BHD: multiple bilateral and basally located lung cysts on a chest CT. Patients exhibiting cysts with other apparent causes were excluded. 

### 2.4. CT Protocol and Analysis

A chest CT was performed using either a 64-slice multidetector CT (MDCT) scanner (Somatom Sensation 64; Siemens Medical Solutions, Erlangen, Germany) or a 128-slice MDCT scanner (Somatom Sensation AS+; Siemens Medical Solutions, Erlangen, Germany or Ingenuity Core 128, Philips Healthcare, Cleveland, OH, USA) according to standard protocol [5]. After acquiring the scout image to determine the field of view, conventional CT scanning was performed with a 1–3 mm reconstruction interval in the mediastinal window setting. The exposure parameters for the CT scans were: 120 kVp, 100–200 mA, and 1–3 mm slice thickness. Image reconstruction for conventional CT scans was performed using the scanner workstation. All CT images were retrieved using a picture archiving and communication system (PACS) (Centricity 4.0; GE Medical Systems, Mountain Prospect, Chicago, IL, USA). Two radiologists (CHP and THK) with >10 years of experience in chest radiology interpretation assessed the CT images. Cyst morphology was categorized as round, oval, or irregular, and irregularity of shape was defined as the presence of all three types of cyst morphologies in one lung. Other variables (number of lung cysts > 40 and maximum diameter) were selected based on previous studies [5,9]. 

### 2.5. NGS

The analysis of gene mutation in FLCN, TSC1, TSC2, CFTR, EFEMP2, ELN, FBLN5, LTBP4, and SERPINA1 was conducted using NGS with the NextSeq 550 System (Illumina, NGS Wet process: ver. Illumina NGS-E2-20201015; Appendix A). Sequencing data from the NextSeq 550 system were aligned to the hg19 human reference genome. Called variants were annotated with the ANNOVAR (http://www.openbioinformatics.org/annovar/) (accessed on 1 June 2023) and the Variant Effect Predictor (http://asia.ensembl.org/info/docs/tools/vep/index.html) (accessed on 1 June 2023). All identified variants were classified as pathogenic, likely pathogenic, and uncertain significance based on the standards and guidelines outlined by the American College of Medical Genetics (Genet Med. 2015; 17:405-24) [10].

### 2.6. Other Tests

Pulmonary function tests were performed to determine whether pulmonary function was impaired using MS-IOS (Masterlab-IOS, Jaeger, Wurzburg, Germany), according to the recommendations of the American Thoracic Society/European Respiratory Society, as reported earlier [11,12]. All subjects consulted with a dermatologist (SEL) who carefully performed a full-body skin examination to check for skin manifestations. Any lesion suspected to be associated with BHD was biopsied for pathological analysis. 

### 2.7. Systematic Review to Recruit BHD Subjects in Korea

We systematically reviewed studies which reported BHD cases in South Korea in accordance with the PRISMA guidelines [13]. We comprehensively searched (1) Web of Science (all databases); (2) Scopus; (3) SPORTDiscus; (4) PubMed; and (5) Cochrane library (Cochrane Database of Systematic Reviews) using the “Birt–Hogg–Dube syndrome” and “Korea” search strings and no restriction regarding the year of publication. The inclusion criteria were: (1) reporting case(s) with BHD syndrome in South Korea and (2) reporting case(s) with a history of pneumothorax, skin manifestations, and renal tumors. The exclusion criteria were: (1) studies not based on humans and (2) studies involving previously reported populations. Two authors (HJP and YJC) independently screened the results to determine relevant studies. Additionally, they reviewed the full-text versions of relevant studies to identify articles that met the above-mentioned criteria. The data were then extracted and analyzed.

**Figure 1:**
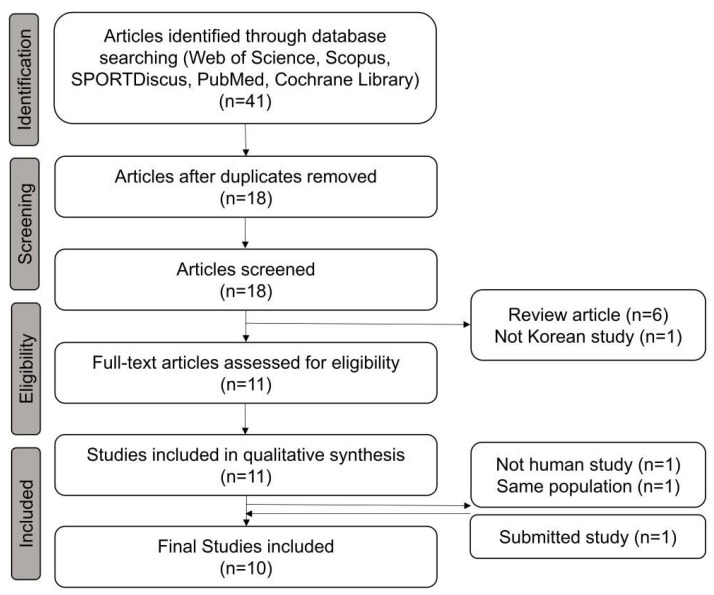
Preferred Reporting Items for Systematic Reviews and Meta-Analyses (PRISMA) flow diagram for the study.

We identified 41 articles and removed 23 duplicate articles. After excluding review articles (*n* = 6) and a study written in Russian (*n* = 1), we excluded two more studies (one experimental and another including a previously reported population). One of the in-press studies conducted at our institution met the inclusion criteria and was included in the final analysis. Finally, ten studies were included in the meta-analysis (Figure 1).

### 2.8. Statistical Analysis

Between-group comparisons of categorical variables were performed using the chi-square or Fisher’s exact test. Based on the Shapiro–Wilk test, parametric and non-parametric continuous variables were compared using an independent two-sample *t*-test and Kruskal–Wallis rank-sum test, respectively. Associations between variables were analyzed using logistic regression analysis. The stepwise regression method was employed, including forward, backward, and bidirectional approaches, to select variables for the multivariate prediction model. Statistical significance was set at *p* < 0.05. ROC curve analysis was performed to identify the ability of the multivariate prediction models to aid BHD diagnosis. The AUC was calculated to assess the sensitivity, specificity, and positive and negative predictive values of the prediction models. The optimal cutoff value was defined using Youden’s J statistic. 

Statistical analyses were conducted using R software (version 4.0.2; R Foundation for Statistical Computing, Vienna, Austria). Spirometry values were converted to Z-scores using reference values from the Global Lung Function Initiative 2012 using the “Rspiro” R package. Logistic regression analysis was performed using the “stats” R package. Survival analysis for Kaplan–Meier curves was performed using the “survival” and “survminer” R packages. ROC analysis was performed using the “ROCR” and “pROC” R packages. The nomogram for the prediction model was drawn using “rms” R package.

## 3. Results

### 3.1. Clinical and Radiological Characteristics 

Based on next-generation sequencing (NGS) analysis, 11 of the 31 (36.7%) enrolled patients were diagnosed with BHD according to the presence of *FLCN* gene mutations. The patients in the BHD group (mean age: 42.2 years) were significantly younger than those in the non-BHD group (mean age: 52.5 years, mean difference [MD] [95% confidence interval], 10.3 [0.9–19.7] years, *p* = 0.040; Table 1). Body mass index, height, weight, sex predominance, respiratory symptoms, and lung function did not differ significantly between the two groups. Pneumothorax was significantly more predominant in the BHD group than in the non-BHD group (90.9% vs. 30.0%, odds ratio [OR] [95% confidence interval], 20.8 [2.2–1074.8], *p* = 0.002). Renal tumors were not observed in the BHD group, and only one in the non-BHD group had a history of renal tumors.

Radiological features of lung cysts on chest computed tomography (CT) differed between the two groups. In the non-BHD group, >40 lung cysts were observed in 65.0% of the study subjects. However, only two (18.2%) subjects had >40 lung cysts in the BHD group (*p* = 0.023). The mean diameters of the largest lung cyst in the BHD and non-BHD groups were 3.3 and 1.9 cm, respectively (MD −1.4 [−2.6–−0.2] cm, *p* = 0.031). Irregularly shaped lesions were present in 81.8% of patients with BHD syndrome and 35.0% of those without BHD syndrome *(p* = 0.023) (Table 1).

### 3.2. Comparison of BHD between Korea and the Rest of the World

We systematically reviewed previous studies in accordance with the Preferred Reporting Items for Systematic Reviews and Meta-Analyses (PRISMA) guidelines, including six case reports [14,15,16,17,18,19] and four original articles [5,6,7,9] (Figure 1). Almost all studies enrolled BHD subjects from specific institutions. However, a study by Park et al. (2022) [6] enrolled patients with BHD by using nationwide claims data using Disease-10 codes, which may have resulted in an overlap of some subjects with those in other studies [6]. In total, 45 patients with BHD (except for those included in the study by Park et al. (2022) [6]) were included in the analysis. Among them, thirty-three (73.3%) patients had a history of pneumothorax, seven (15.6%) had skin manifestations, and four (8.9%) had renal tumors. Part et al. (2022) described a similar prevalence of these conditions (65.4%, 16.0%, and 11.5%, respectively), although a recent worldwide meta-analysis reported on a prevalence of 50.9%, 47.9%, and 22.5%, respectively [20]. Pneumothorax was more prevalent in South Korean patients with BHD (OR, 2.66 [1.36–5.20], *p* = 0.003); however, skin manifestations and renal tumors were relatively rare in South Korean patients with BHD in comparison with those of other nationalities (OR, 0.20 [0.09–0.45], *p* < 0.001; and 0.34 [0.12–0.95], *p* = 0.027, respectively) (Table 2). 

### 3.3. Prediction Model

**Figure 2:**
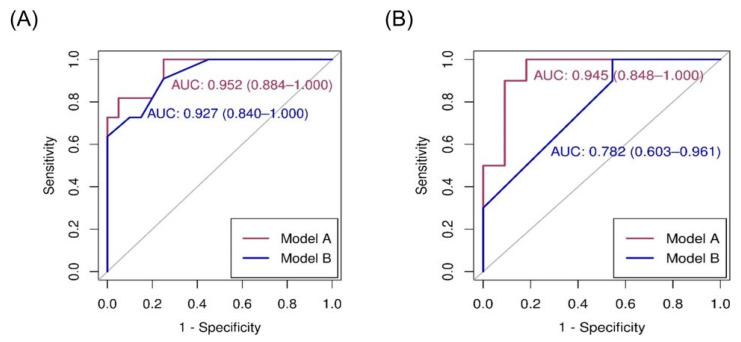
Area under the curve (AUC) of receiver operating characteristic (ROC) for diagnosis of BHD in cohort (**A**) and external validation (**B**). BHD: Birt–Hogg–Dube syndrome.

Details of logistic regression and receiver operating characteristic (ROC) analyses between variables and *FLCN* mutations are presented in Table 3. Using univariate analysis, chest pain, history of pneumothorax, and radiological features (number, maximal diameter, and shape of lung cysts) were significantly associated with *FLCN* mutations. Skin and renal lesions were not significant predictors of BHD. Prediction models A and B were selected using forward and stepwise selection, respectively (Table 3). Prediction model A was based on age, number of lung cysts, and a maximal lung cyst diameter of >2 cm. The ROC analysis of model A for the diagnosis of BHD was 0.952 (95% confidence interval [CI], 0.884–1.000). The sensitivity at Youden’s index was 0.818, while the specificity was 0.950. Prediction model B was based on a history of pneumothorax, number of lung cysts of >40, and irregularity in the lung cyst shape. The ROC analysis of model B for the diagnosis of BHD was 0.927 (95% CI, 0.840–1.000) (Figure 2A). The sensitivity at Youden’s index was 0.909, while the specificity was 0.750.

**Figure 3:**
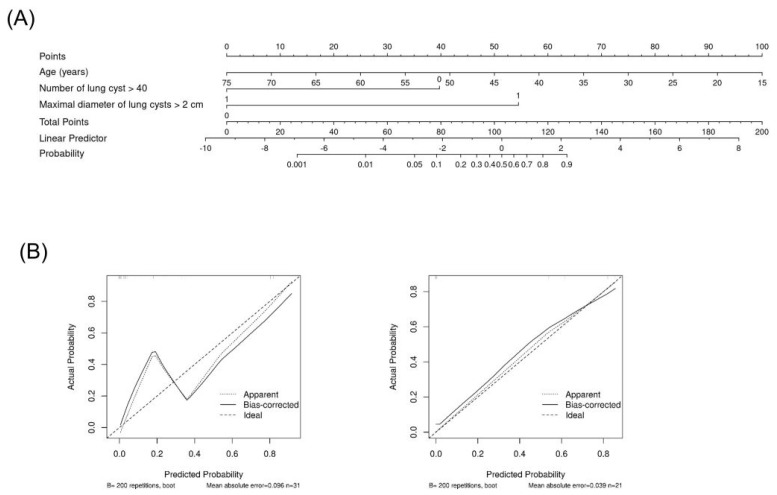
Nomogram based on the prediction model A. Nomogram (**A**) and calibration in cohort (left in (**B**)) and in external validation (right in (**B**)).

When the prediction models were evaluated using the external cohort, model A had a higher predictive power, similar to that of the internal validation (area under the curve [AUC] of ROC [95% CI]: 0.945 [0.848–1.000]), whereas model B had a lower predictive power (AUC of ROC 0.782 [0.603–0.961]; Figure 2B). The nomogram based on prediction model A for clinical use is shown in Figure 3. 

### 3.4. NGS Results of the Study Subjects

In the BHD group, we identified three novel and previously unreported mutations: c.1098G>A, c.139G>T, and c.1335del. In addition, two patients have missense mutations in the CFTR gene (Lys411Glu and Glu217Gly), and one patient has a missense mutation in the TSC2 gene (Met740Thr). In the non-BHD group, nine subjects demonstrated other lung cyst-related gene mutations: missense mutations of gln1352His (*CFTR*), c.849-8A>G (*TSC2*), p.Leu571_Gly579del (*ELN*), p.Gly1142= (*LTBP4*), Ile125Thr (*CFTR*), Arg692Gln (*TSC1*), Ala496Thr (*LTBP4*), Arg31Cys (*CFTR*), Gly172Arg (*SERPINA1*), Leu1306Phe (*LTBP4*), and Asp192= (*TSC2*) (Table 4).

## 4. Discussion

To the best of our knowledge, this study included the first systematic review of South Korean patients with BHD. We found that South Korean patients with BHD had a higher prevalence of history of pneumothorax and a lower prevalence of skin and renal lesions than those from other countries. Global data suggest that patients with BHD frequently present with typical skin lesions (84%) and renal cell carcinoma (19–35%) [21,22,23]. However, in this study, fewer South Korean patients with BHD presented with skin lesions (15.6–16.0%) and renal cell carcinoma (8.9–11.5%). The prediction model, which included detailed lung cyst characterization but did not include skin and renal lesions, could predict BHD with significant power (AUC: 0.927–0.952). In South Korean patients with BHD, skin and renal lesions are not observed frequently. Therefore, BHD syndrome should be considered in patients with multiple bilateral basal lung cysts, despite the absence of skin or renal lesions.

Interestingly, the prevalence of skin and renal lesions varied according to the study design. Case studies reported a relatively high prevalence of skin manifestations; however, three of six cases were reported by the same dermatologist, which may have resulted in selection bias. Skin lesions were not reported by Park et al. [5] and Choi et al. [9], similar to this study. The low prevalence of skin lesions may raise questions regarding the performance of the skin test. In the above-mentioned studies, dermatologists carefully performed full-body skin examinations, and biopsies were performed as necessary. However, the pathological findings did not match the typical skin lesions observed in BHD. A prospective study also reported a low prevalence of skin lesions, and Park et al. (2022) [6] reported similar values to those mentioned in nine other South Korean studies. However, Park et al. (2022) [6] did not include all the reported South Korean cases (only 26 subjects, not more than 45 subjects), which might be due to the limitation of including subjects with BHD based only on the diagnostic code that may have been omitted in some cases.

Detailed characteristics of lung cysts on chest CT scans were critical for predicting BHD in our study. Classical diagnostic criteria suggest that multiple bilateral basal lung cysts may indicate BHD. However, the number of lung cysts, a lung cyst diameter of >2 cm, and an irregular cyst shape were also important characteristics to differentiate BHD in this study. Incidental lung cysts are frequently observed in the healthy population (7.6–25%) [24,25], and several incidental lung cysts (<40) might be part of the normal aging process [25]. The *FLCN* gene, which is mutated in BHD, plays an essential role in epithelial cell integrity and lung homeostasis [26]. It is required for lung alveolar epithelial cell survival and modulates E-cadherin, LKB1, and AMPK activation [26]. *FLCN* loss leads to the development of multiple irregular-shaped lung cysts in the basilar and subpleural regions of the lungs (including the visceral pleural and interlobular fissure) [27,28]. We propose that a more detailed description, beyond the classical diagnostic criteria, is needed to differentiate BHD accurately.

This study reports three novel mutations and NGS results beyond the *FLCN* gene, c.1098G>A, c.139G>T, and c.1335del. Moreover, we identified mutations associated with other lung cystic diseases. Some studies have attempted to conduct NGS to identify other associated genes [29,30]. If more data are obtained, it may be possible to determine the effects of other associated genes on lung cysts. Nevertheless, the findings of this study will be helpful for future NGS-based studies in patients with BHD.

Our study findings suggest that BHD should be considered in South Korean subjects with multiple bilateral basal lung cysts, even if skin or renal lesions are absent. Rather than skin manifestations and renal lesions, a personal or family history of pneumothorax may be more indicative of BHD in South Korean populations. Notably, family screening is required when a patient is diagnosed with BHD as this disease is associated with breast, colorectal, lung, and skin cancers [23,31,32]. Thus, early, active cancer screening is essential for patients with BHD and their families. In addition, the development of pneumothorax should be monitored.

This study has some limitations. First, it involved a small number of subjects. In addition, we conducted a screening of patients with multiple lung cysts, specifically focusing on individuals who underwent chest CT scans rather than the general population. Furthermore, we included only patients who met one of the minor criteria for BHD, which involves the presence of multiple bilateral and basally located lung cysts. Consequently, there may be potential selection bias associated with this approach. Second, we did not systematically review the global BHD data. A well-designed meta-analysis should be conducted. Finally, we could not find a significant correlation between other cystic lung disease-associated gene mutations and clinical or radiological findings.

## 5. Conclusions

The features of BHD in South Korean patients are different from those reported in patients of other countries, with skin and renal lesions being rarely present. Detailed characterization of lung cysts is necessary to define BHD in South Korean patients, even if skin or renal lesions are absent. Further studies are required to confirm the clinical significance of other lung disease-associated gene mutations.

## Figures and Tables

**Table 1:** Clinical and radiologic characteristics of subjects enrolled in this study.

	Total	Non-BHD	BHD	Difference (95% CI) ^†^	*p*-Value
**Clinical features**	**(*N* = 31)**	**(*N* = 20)**	**(*N* = 11)**		
Age (year)	48.8 ± 13.5	52.5 ± 12.9	42.2 ± 12.6	10.3 (0.9–19.7)	0.040
BMI (kg/m^2^)	23.0 ± 3.7	22.7 ± 3.7	23.4 ± 3.9	−0.7 (−3.5–2.1)	0.640
**Sex**				0.24 (0.0–2.5)	0.377
Male	7 (22.6%)	6 (30.0%)	1 (9.1%)		0.372
Female	24 (77.4%)	14 (70.0%)	10 (90.9%)		
**Symptoms**					
Cough	3 (9.7%)	1 (5.0%)	2 (18.2%)	4.0 (0.2–261.4)	0.281
Chest pain	7 (22.6%)	2 (10.0%)	5 (45.5%)	6.9 (0.9–91.4)	0.067
Hemoptysis	0 (0.0%)	0 (0.0%)	0 (0.0%)		0.999
**Pneumothorax**	16 (51.6%)	6 (30.0%)	10 (90.9%)	20.8 (2.2–1074.8)	0.002
Age at first event (yr)	37.3 ± 13.5	40.5 ± 14.4	35.4 ± 13.3	5.1 (−5.2–15.4)	0.484
Surgical intervention	12 (75.0%)	4 (66.7%)	8 (80.0%)	1.91 (0.1–36.4)	0.604
Familiar history	3 (9.7%)	0 (0.0%)	3 (27.3%)	Inf (0.3–Inf)	0.250
**Renal tumor**	1 (3.2%)	1 (5.0%)	0 (0.0%)	0 (0.0–70.8)	0.999
**Lung function**	**(*N* = 16)**	**(*N* = 10)**	**(*N* = 6)**		0.999
FVC (liter)	3.3 ± 1.0	3.3 ± 1.1	3.2 ± 1.1	0.1 (−1–1.2)	0.900
FVC (%, reference)	93.7 ± 16.4	94.2 ± 17.8	92.8 ± 15.3	1.4 (−15.8–18.6)	0.878
FVC (z-score)	−0.3 ± 1.5	−0.1 ± 1.6	−0.7 ± 1.2	0.6 (−0.9–2.1)	0.465
FEV_1_ (liter)	2.5 ± 0.9	2.4 ± 1.0	2.6 ± 0.8	−0.2 (−1.1–0.7)	0.785
FEV_1_ (%, reference)	89.4 ± 17.2	87.8 ± 18.7	92.0 ± 15.7	−4.2 (−22.1–13.7)	0.652
FEV_1_ (z-score)	−0.8 ± 1.3	−0.8 ± 1.5	−0.9 ± 0.9	0.1 (−1.2–1.4)	0.847
FEV_1_/FVC (%)	75.5 ± 8.8	72.7 ± 9.3	80.2 ± 6.0	−7.5 (−15.9–0.9)	0.102
FEV_1_/FVC (z-score)	−1.0 ± 1.3	−1.3 ± 1.4	−0.4 ± 1.0	−0.9 (−2.2–0.4)	0.210
FEF_25–75%_ (liter/sec)	2.2 ± 1.4	2.1 ± 1.6	2.5 ± 0.8	−0.4 (−1.8–1)	0.578
FEF_25–75%_ (%, REF)	72.2 ± 32.7	66.8 ± 39.5	81.3 ± 15.6	−14.5 (−47.9–18.9)	0.408
FEF_25–75%_ (z-score)	−0.7 ± 1.3	−0.9 ± 1.5	−0.4 ± 0.8	−0.5 (−1.8–0.8)	0.412
DLCO (%, REF)	85.5 ± 19.7	88.9 ± 23.6	79.8 ± 10.6	9.1 (−11.1–29.3)	0.491
DLCO/VA (%, REF)	100.0 ± 14.2	98.9 ± 15.6	102.0 ± 13.3	−3.1 (−18.1–11.9)	0.744
**Radiologic features of lung cysts on chest CT**	**(*N* = 31)**	**(*N* = 20)**	**(*N* = 11)**		
Number (above 40)	15 (48.4%)	13 (65.0%)	2 (18.2%)	0.1 (0.0–0.9)	0.023
Maximal diameter (cm)	2.4 ± 1.8	1.9 ± 1.4	3.3 ± 2.0	−1.4 (−2.6–−0.2)	0.031
Irregularity of shape	16 (51.6%)	7 (35.0%)	9 (81.8%)	7.8 (1.2–93.5)	0.023

Data are presented as mean ± standard deviation. CI: confidence interval; BHD: Birt–Hogg–Dube syndrome; BMI: Body mass index; FVC: forced vital capacity; FEV_1_: forced expiratory volume in 1 s; FEF_25–75%_; forced expiratory flow between 25% and 75% of vital capacity; DLCO: diffusing capacity of the lung for carbon dioxide; VA: volume of alveolar; REF: reference. ^†^ For continuous variables, we reported the mean difference, while for categorical variables, we reported the odds ratio.

**Table 2:** Comparison of BHD between Korea (obtained from systematic review) and rest of the world.

Study	Number	History of Pneumothorax	Skin Manifestation	Renal Tumor
This study	11	10 (90.9%)	0 (0.0%)	0 (0.0%)
6 Cases (2008–2021) [14,15,16,17,18,19]	6	5 (83.3%)	4 (66.7%)	0 (0.0%)
Park et al. in 2017 [5]	6	3 (50.0%)	0 (0.0%)	1 (16.7%)
Lee et al. in 2019 [7]	12	8 (66.7%)	3 (25.0%)	3 (25.0%)
Choi et al. in 2023 [9]	10	7 (70.0%)	0 (0.0%)	0 (0.0%)
**Total in Korea**	**45**	**33 (73.3%)**	**7 (15.6%)**	**4 (8.9%)**
Park et al. in 2022 (cover total Korea) [6]	26	17 (65.4%)	4 (16.0%)	3 (11.5%)
**Worldwide meta-analysis cohort in 2021**	**1038**	**528/1038 (50.9%)**	**474/989 (47.9%)**	**209/929 (22.5%)**
Difference (95% confidence interval) between study in Korea and worldwide meta-analysis (between gray color groups)		2.66 (1.36–5.20)	0.20 (0.09–0.45)	0.34 (0.12–0.95)
*p*-value for comparison between study in Korea and worldwide meta-analysis (between gray color groups)		0.003	<0.001	0.027

**Table 3:** Prediction model of BHD in patients with multiple lung cysts.

Univariate Analysis				
Variables	Coefficients	*p*-value	AUC of ROC [95% CI]	
Age (year)	−0.064	0.053	0.730 [0.535–0.924]	
BMI (kg/m^2^)	0.050	0.627	0.548 [0.323–0.773]	
Sex	−1.455	0.208	0.605 [0.468–0.741]	
Cough	1.440	0.264	0.566 [0.437–0.695]	
Chest pain	2.015	0.036	0.677 [0.509–0.846]	
History of pneumothorax	3.150	0.006	0.805 [0.668–0.941]	
Number of lung cyst (above 40)	−2.123	0.020	0.734 [0.574–0.895]	
Maximal diameter of lung cyst (cm)	0.484	0.050	0.757 [0.562–0.952]	
Irregularity of lung cyst shape	2.123	0.020	0.764 [0.599–0.928]	
Maximal diameter of lung cysts > 2 cm	2.367	0.007	0.734 [0.574–0.895]	
**Multivariate analysis**				
	Coefficients	*p*-value	AUC of ROC [95% CI]	VIF
Prediction model A (multivariate analysis)				
Age (year)	−0.151	0.029		2.134
Number of lung cyst (above 40)	−3.590	0.033	0.952 [0.884–1.000]	1.638
Maximal diameter of lung cysts > 2 cm	4.923	0.012		2.355
Prediction model B (multivariate analysis)				
History of pneumothorax	2.818	0.043		1.056
Number of lung cyst (above 40)	−3.108	0.033	0.927 [0.840–1.000]	1.51
Irregularity of lung cyst shape	2.719	0.060		1.446

BMI: body mass index; AUC: area under the curve; ROC: receiver operating characteristic; CI confidence interval; VIF: variance inflation factor.

**Table 4:** Results of next-generation sequencing performed on study subject samples.

No.	Location	Variant (cDNA)	Protein Change	Type of Mutation	Reference of Pathologic Mutation (PMID)	Other Gene Mutations ^†^
1	Exon 11	c.1277dupT	p.His429Profs*27	Frame shift	25519458	
2	Exon 13	c.1522_1524del	p.Lys508del	Inframe deletion	19659657	
3	Exon 12	c.1429C>T	p.Arg477*	Nonsense	30360018	
4	Exon 10	c.1098G>A	p.Trp366*	Nonsense	**Novel mutation**	TSC2: missense Met740Thr
5	Exon 6	c.469_471del	p.Phe157del	Inframe deletion	18505456, 21538689	CFTR: missense Lys411Glu
6	Exon 4	c.139G>T	p.Glu47*	Nonsense	**Novel mutation**	
7	Exon 6	c.469_471del	p.Phe157del	Inframe deletion	18505456, 21538689	
8	Exon 11	c.1285dup	p.His429Profs*27	Frame shift	12471204	
9	Exon 14	c.1557del	p.Phe519Leufs*18	Frame shift	18437022, 30360018	
10	Exon 12	c.1335del	p.Arg446Valfs*22	Frame shift	**Novel mutation**	
11	Intron 13	c.1539-2A>G	p.?	Splice	27652079	CFTR: missense Glu217Gly
12	FLCN mutations were not identified.					CFTR: missense Gln1352His
13						TSC2: c.849-8A>G
14						ELN: p.Leu571_Gly579del
15						LTBP4: p.Gly1142=
16						CFTR: missense Ile125Thr
17						TSC1: missense Arg692Gln LTBP4: missense Ala496Thr
18						CFTR: missense Arg31Cys SERPINA1: missense Gly172Arg
19						LTBP4: missense Leu1306Phe
20						TSC2:Asp192=
21 to 31	No mutations were identified in the FLCN, TSC1, TSC2, CFTR, EFEMP2, ELN, FBLN5, LTBP4, and SERPINA1 genes.					

^†^ TSC1, TSC2, CFTR, EFEMP2, ELN, FBLN5, LTBP4, and SERPINA1 genes.

## Data Availability

The datasets used and analyzed during the current study are available from the corresponding author on reasonable request.

## Abbreviations

AUC	area under the curve
BHD	Birt–Hogg–Dube syndrome
CI	confidence interval
CT	computed tomography
FLCN	folliculin gene
NGS	next-generation sequencing
PCR	polymerase chain reaction
PRISMA	Preferred Reporting Items for Systematic Reviews and Meta-Analyses
ROC	receiver operating characteristic

## Appendix A. Genelists for NGS

***Gene Name***	***OMIM***	***Location***	***Reference cDNA***	***OMIM***	***Inheritence***
*FLCN*	*607273*	*17p11.2*	*NM_144997.5*	*Birt–Hogg–Dube syndrome*	*AD*
*TSC1*	*605284*	*9q34.13*	*NM_000368.4*	*Lymphangioleiomyomatosis*	*AD*
*TSC2*	*191092*	*16p13.3*	*NM_000548.3*	*Lymphangioleiomyomatosis, somatic*	*AD*
*CFTR*	*602421*	*7q31.2*	*NM_000492.3*	*Congenital bilateral absence of vas deferens*	*AR*
*EFEMP2*	*604633*	*11q13.1*	*NM_016938.4*	*Cutis laxa, autosomal recessive, type IB*	*AR*
*ELN*	*130160*	*7q11.23*	*NM_001278939.1*	*Cutis laxa, AD*	*AD*
*FBLN5*	*604580*	*14q32.12*	*NM_006329.3*	*Cutis laxa, autosomal dominant 2*	*AR/AD*
*LTBP4*	*604710*	*19q13.2*	*NM_003573.2*	*Cutis laxa, autosomal recessive, type IC*	*AR*
*SERPINA1*	*107400*	*14q32.13*	*NM_000295.4*	*Emphysema due to AAT deficiency*	*AR*
